# Expression of Monocytes Subsets in Patients Diagnosed With Coronary Artery Atherosclerosis and Their Impact on Disease Severity

**DOI:** 10.7759/cureus.74670

**Published:** 2024-11-28

**Authors:** Asmaa Khalf Kamel, Naglaa M Farag, Emad Allam, Mohamed Khaled, Doaa Elzaeem Ismail

**Affiliations:** 1 Department of Clinical Pathology, Minia University Faculty of Medicine, Minia, EGY; 2 Department of Cardiology, Minia University Faculty of Medicine, Minia, EGY

**Keywords:** atherosclerosis, cd16+ monocytes, flow cytometery, lipoprotein (a), non classical monocytes

## Abstract

Introduction

Many studies have supported inflammation as a mediator of lipoprotein (a) (Lp(a)) induced increase in cardiovascular disease risk, as it has pro-inflammatory effects on endothelial cells and monocytes.

Aim

This study aims to correlate Lp(a) level with different monocyte subsets in coronary atherosclerotic patients with different severity.

Method

The study included 60 patients with a mean age of 53.1 ± 10.5 diagnosed as coronary atherosclerotic patients by coronary angiography. Lp levels were measured using enzyme-linked immunosorbent assay (ELISA), while blood counts and monocyte subsets were analyzed by flow cytometry, and 30 apparently healthy individuals were included as the control group.

Results

Patients showed significantly higher median monocytic %, Lp(a), and higher C-reactive protein (CRP) values than the control group. Patients were subdivided into two groups: normal Lp(a) < 6.2 mg/dL (n = 24) and hyperlipoproteinemia(a) (hyper Lp(a)) ≥ 6.2 mg/dL (n = 36). Patients with hyper Lp(a) had higher non-classical monocytes (31.5% vs. 20%). Coronary atherosclerosis severity was associated with higher Lp(a) levels as well as non-classical monocytes; patients with mild atherosclerosis showed the highest classical and intermediate subset levels. While for a non-classical subset, patients with severe atherosclerosis showed the highest median level. A significant moderate positive correlation between Lp(a) and monocyte counts, as well as monocyte-lymphocyte (M/L) index and non-classical monocytes, was found.

Conclusions

Hyper Lp(a) and increased count of non-classical monocytes are significantly increased with disease progression (triple-vessel coronary disease risk). These results suggest that the expansion of non-classical monocytes is a cardiovascular disease (CVD) risk and predictor for disease severity. Strategies targeting inflammatory monocytes may slow CVD progression.

## Introduction

According to the World Health Organization, cardiovascular disease (CVD) is a leading cause of death globally, with a mortality of 17.9 million per year [1]. Clinical presentations range from asymptomatic to stable angina, acute coronary syndrome, heart failure, and sudden cardiac death [2]. Monocytes play a crucial role in the local inflammatory response, plaque development, and thrombus formation [3,4]. Few studies have been conducted in humans, making monocytes attractive therapeutic targets [3,4]. Monocyte subsets (classical monocyte (CM), intermediate monocyte (IM), and non-classical monocyte (NCM)) differentially contribute to atherosclerosis pathogenesis. Patients with CVD have a higher percentage of IM and NCM than controls [5].

Risk-factor modification to prevent or reverse atherosclerosis progression can provide benefits at all stages of atherosclerotic disease, even as secondary prevention. Preventive therapies, including lipid-lowering, are imperfect. Reprogramming macrophages to an anti-inflammatory phenotype can promote plaque stabilization and atherosclerotic cardiovascular disease (ASCVD) regression [4]. Setting a cutoff can determine whether to stent the diseased segment or not and can also help determine which patients would benefit from intervention and medical therapy [6].

Lipoprotein (a) (Lp(a)) levels are controlled by production rather than catabolism. Each individual inherits and expresses two copies of Lp(a), one from each parent, unless homozygous for two Lp(a) genes [7]. Hyperlipoproteinemia(a) (hyper Lp(a)) occurs in 20% of the population and is the most common genetic lipid disorder. It is higher in calcific aortic valve disease (CAVD) and chronic kidney disease (CKD) [8]. Because of such high CVD risk, recent lipid guidelines stress evaluating Lp(a) to stratify CVD risk [9]. Large genetic epidemiologic studies have renewed interest in Lp(a) by finding strong genetic evidence of the associations of high Lp(a) with increased risk of coronary heart disease (CHD), heart failure, and mortality [10]. CAD patient prognosis, especially in those with acute myocardial ischemia, has significantly improved due to advancements in technical coronary interventions, stents, and further technical evolution [11].

Some studies reported that genetic lowering of Lp(a) by 10 mg/dL is associated with a 5.8% lower risk of CHD [7]. Recent studies have reported conflicting results regarding measuring monocyte subgroups as a marker for inflammatory activation and promotion of atherogenesis [5]. The aim of this study is to investigate the association between increased plasma Lp(a) level and monocyte subsets in patients with different coronary atherosclerosis severity.

## Materials and methods

The study included 60 coronary artery atherosclerosis patients diagnosed by angiography and on treatment (group I), selected from the Cardiology Clinic, Faculty of Medicine, Minia University Hospital, Minia, Egypt, and 30 apparently healthy individuals (group II) with matched age and sex as a control group. Group I was subdivided into three subgroups according to disease severity and number of affected vessels in angiography: group Ia: mild cases, who have a single vessel affected; group Ib: moderate cases, having double vessels affected; and group Ic: severe cases, having multiple vessels affected.

Patients with the following conditions were excluded from the study: Lp(a) lowering therapy, acute coronary syndrome, congestive heart failure, acute inflammatory or infectious diseases in the last three months, connective tissue disease, severe thyroid dysfunction, CKD stage IV or V, and liver disease.

The study had local Research Ethics approval of Minia University, Faculty of Medicine, Institutional Review Board "MUFMIRB" (approval no. 133:11/2021), and written consent was obtained from patients or their legal guardians.

Both patients and control groups were subjected to the following: full history-taking, thorough clinical examination, and laboratory investigations. These investigations included a complete blood count (CBC), monocyte lymphocyte index, CD14 and CD16 assay by flow cytometry, C-reactive protein (CRP), liver and renal function, and lipid profile. The level of low-density lipoprotein cholesterol (LDL-C) was calculated using Friedewald’s formula:

LDL-C (mg/dL) = TC (mg/dL) − HDL-C (mg/dL) − TG (mg/dL)/5.

Lp(a) assay by enzyme-linked immunosorbent assay (ELISA), using monospecific polyclonal sheep antibodies against human Lp(a). The kit was supplied by Biokit for scientific research in China. The minimum detectable limit of Lp(a) is less than 0.09 ng/mL. This assay has high sensitivity, excellent specificity, and no significant cross-reactivity or interference between Lp(a) and its analogs.

Lymphocyte-monocyte index was calculated as the ratio of absolute lymphocyte count to absolute monocyte count. Direct immunofluorescence was used to assess monocyte immunophenotyping using fluorescently labeled antibodies to CD14 and CD16 antigens. The kit was supplied by Kemet Medical. Readings were obtained using flow cytometry (BD-FACSCanto II Flow Cytometer, Becton, Dickinson and Company, Franklin Lakes, NJ) following the manufacturer’s manuals. Gating of monocytes was performed using forward scatter parameters, and monocyte subsets were identified as classical (CD14++CD16−), intermediate (CD14++CD16+), and non-classical (CD14+CD16++) using flow cytometry. BD FACSDiva (Data-Interpolating Variational Analysis, Paris, France) software was used for data processing.

Results were expressed as percentages of cells positive for CD14 and CD16. A case was considered positive for CD14 or CD16 expression if the percentage exceeded the cutoff of 20%. The study obtained local research ethics approval from the Minia Medical College Ethics Committee, and written consent was obtained from each patient or their legal guardians.

Qualitative data were presented as frequencies and percentages. The chi-square test was used to compare qualitative variables. Quantitative data were explored for normality by checking the distribution of data and using tests of normality (Kolmogorov-Smirnov and Shapiro-Wilk tests). All data showed normal (parametric) distribution except for lipid profile, alanine aminotransferase (ALT), aspartate aminotransferase (AST), total leucocytic counts, monocytes %, monocytes count, monocyte-lymphocyte (M/L) index, Lp(a), and monocytes subset percentages data which showed non-normal (non-parametric) distribution. Parametric data were presented as mean and standard deviation (SD) values, while non-parametric data were presented as median and range values. For parametric data, the Student’s t-test was used for comparisons between the two groups. One-way ANOVA followed by Bonferroni’s post-hoc test was used for comparisons between more than two groups. For non-parametric data, the Mann-Whitney U test was used for comparisons between the two groups. Kruskal-Wallis test, followed by Dunn’s test, was used for comparisons between more than two groups. Spearman’s correlation coefficient was used to determine the significant correlation between Lp(a), monocyte counts, M/L index, and monocyte subsets. The receiver operating characteristic (ROC) curve was constructed to determine the cutoff value for Lp(a) to differentiate between atherosclerosis and normal subjects. ROC curve analysis was performed with MedCalc® Statistical Software version 19.5.1 (MedCalc Software Ltd, Ostend, Belgium; https://www.medcalc.org; 2020). Binary logistic regression analysis was used to determine significant predictors of Lp(a) categories. Model fit was tested using the chi-square test and pseudo-R^2^ tests, and the model was fit to describe the relations between the dependent and independent variables. The significance level was set at p ≤ 0.05. Statistical analysis was performed with IBM SPSS Statistics for Windows Version 23.0 (IBM Corp., Armonk, NY).

## Results

Table 1 presents demographic and laboratory data. Patients showed significantly higher median monocytic percentage and Lp(a) (p = 0.045, <0.001, <0.001), as well as a higher prevalence of positive CRP than the control group (p = 0.026). No significant differences were observed between patients and controls regarding gender, age, and blood picture parameters. 

**Table 1:** Comparison between the studied groups regarding demographic and laboratory data HB: hemoglobin, PLT: platelets, WBCs: white blood cells, M/L index: monocyte-lymphocyte index, Lp(a): lipoprotein (a), CRP: C-reactive protein

Data	Group I (n = 60)	Group II (n = 30)	p-value	Mild (n = 20)	Moderate (n = 20)	Severe (n = 20)	p-value
Age (year)	53.1 ± 10.5	49 ± 10.3	0.087	54.2 ± 8	55.2 ± 10	49.9 ± 12.7	0.236
Gender (male, female)	35 (58.3), 25 (41.7)	17 (65.7), 13(43.3)	0.880	11 (55), 9 (45)	14 (70), 6 (30)	10 (50), 10 (50)	0.410
HB (g/dL)	13.23 ± 1.75	13.43 ± 1.41	0.588	13.34 ± 1.78	13.57 ± 1.77	12.8 ± 1.71	0.363
PLT (× 10^3^/µL)	255.1 ± 85.2	282.4 ± 75.3	0.141	240 ± 74	230.4 ± 75.5	294.9 ± 93.9	0.033*
WBCs (× 10^3^/µL)	6.6 (4-18)	6.95 (4-10.9)	0.292	5.05 (4-10.4)	6.1 (4-18)	7.2 (4.2-11.9)	0.076
Monocyte count (×10^3^/µL)	519 (192-1972)	465 (168-780)	0.336	450 (250-936)	434 (192-1440)	777 (212-1972)	0.008*
Monocytes (%)	8 (3-17)	6 (3-10)	0.045*	8 (5-17)	7 (3-13)	10.5 (4-17)	0.007*
Lymphocytes (%)	33.55 ± 10.04	31.5 ± 6.97	0.319	32.85 ± 5.13	33.05 ± 11.93	34.75 ± 11.91	0.811
Neutrophils (%)	53.95 ± 11.86	55.87 ± 8.91	0.437	55.65 ± 7.29	55.6 ± 12.93	50.6 ± 14.44	0.307
M/L index	0.26 (0.1-0.8)	0.22 (0.1-0.33)	0.063	0.25 (0.14-0.6)	0.22 (0.1-0.4)	0.3 (0.1-0.8)	0.100
Classical (%)	47 (9-74)	80.5 (70-88)	<0.001*	62.5 (44-74)	49 (38-71)	29.5 (9-44)	<0.001*
Intermediate (%)	13.8 (2-37)	7 (2-13)	<0.001*	14 (8-20)	14 (7-26)	8.5 (2-37)	0.005*
Non-classical (%)	38.9 (18.3-90)	11 (6-25)	<0.001*	23.5 (13-40)	35.5 (15-52)	61 (19-87)	<0.001*
Lp(a)	6.75 (1.3-18)	3.65 (2.1-7.4)	<0.001*	6.3 (1.3-13.5)	6.55 (3.9-17)	6.85 (2.5-18)	0.779
CRP positive (%)	40	16.7	0.026*	35	40	45	0.03*

Patients with atherosclerosis exhibited significantly higher median values for IM and NCM subsets compared to the control group (p < 0.001), while CM subsets showed a significant increase in the control group compared to the atherosclerotic group (Table 1).

Association between different variables and severity of atherosclerosis

Platelet and monocyte (count and percentage) showed significant increases in the severe atherosclerotic subgroup compared to mild and moderate subgroups (p = 0.033, 0.008, 0.007, respectively). Other CBC parameters showed no significant difference among atherosclerotic subgroups. There were no significant differences between the three subgroups regarding gender distribution, age, Lp(a), and CRP (Table 1).

With regard to monocyte subsets, there were significant differences between groups. Pair-wise comparisons between the groups revealed that patients with mild atherosclerosis showed the highest classical and intermediate subset levels (p < 0.001, 0.005, respectively). While for the non-classical subset, patients with severe atherosclerosis showed the highest median level (p < 0.001). We previously stated that CMs were more abundant in control subjects than in patients with coronary atherosclerosis. To clarify, we found that the CM subset was higher in controls when compared to all cases combined. However, when we examined cases based on disease severity (mild, moderate, and severe), we observed that the mild subgroup had a higher proportion of CMs compared to the moderate and severe subgroups. Meaning, triple‐vessel CAD was more common in patients having a lower percentage of classical CD14++CD16− and intermediate CD14++CD16+ monocytes and a higher percentage of non‐classical CD14+CD16++ monocytes (Table 1).

Correlation between Lp(a), monocyte counts, M/L index, and monocyte subsets

**Figure 1:**
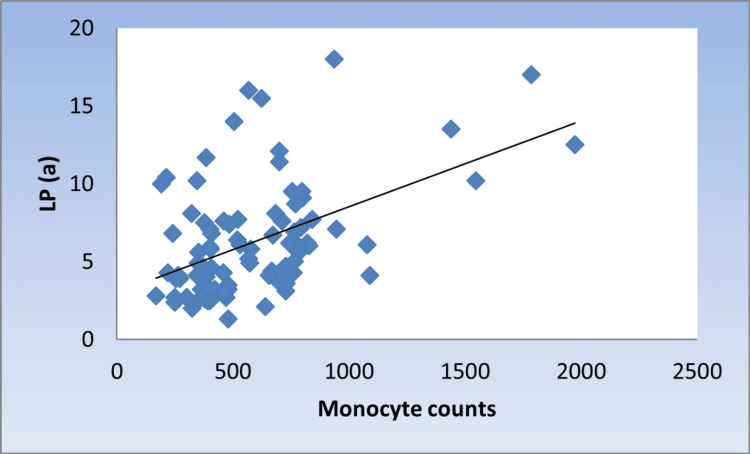
Scatter diagram representing positive correlation between monocyte counts and Lp(a) Lp(a): lipoprotein (a)

**Figure 2:**
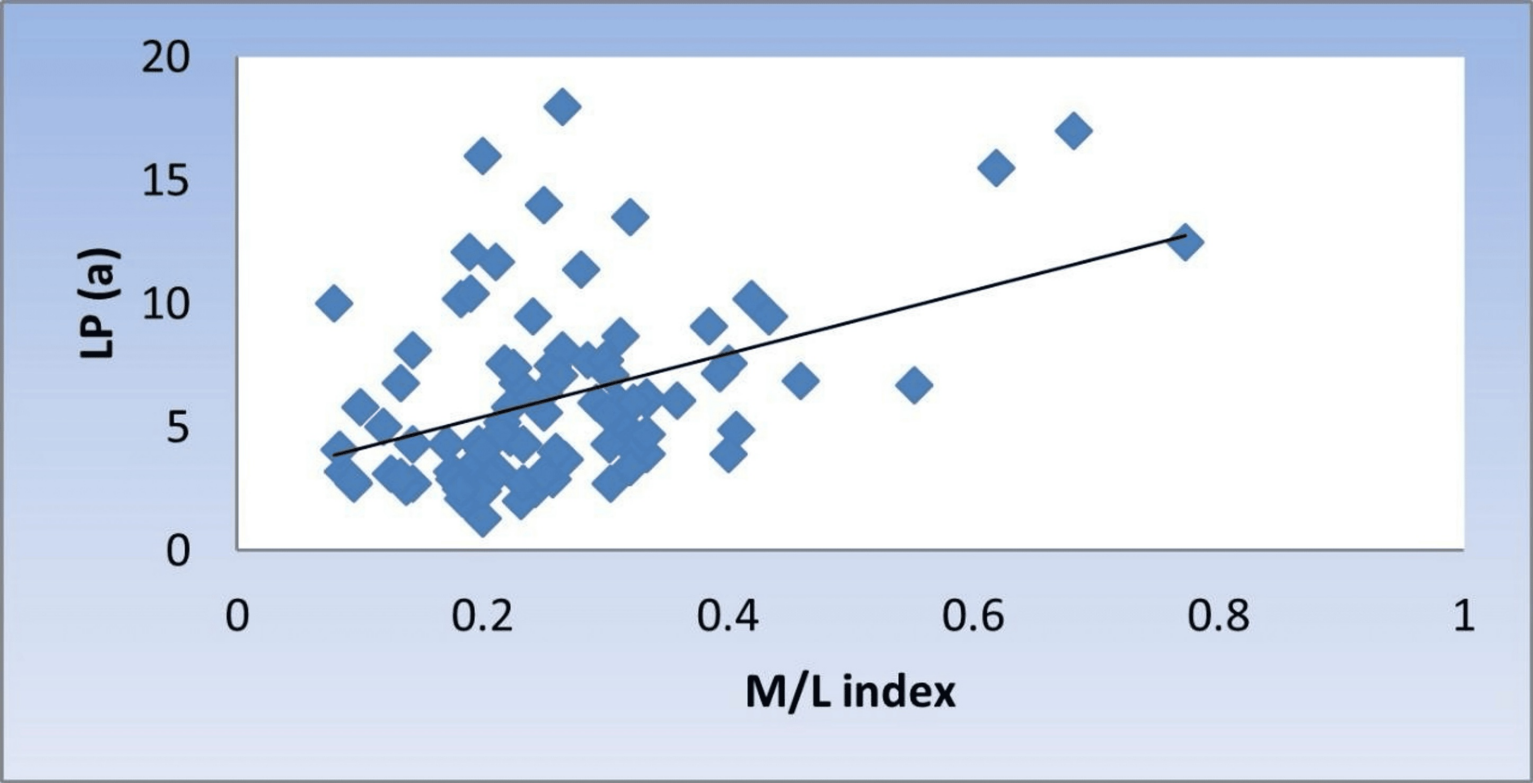
Scatter diagram representing positive correlation between M/L index and Lp(a) Lp(a): lipoprotein (a); M/L: monocyte lymphocyte

**Figure 3:**
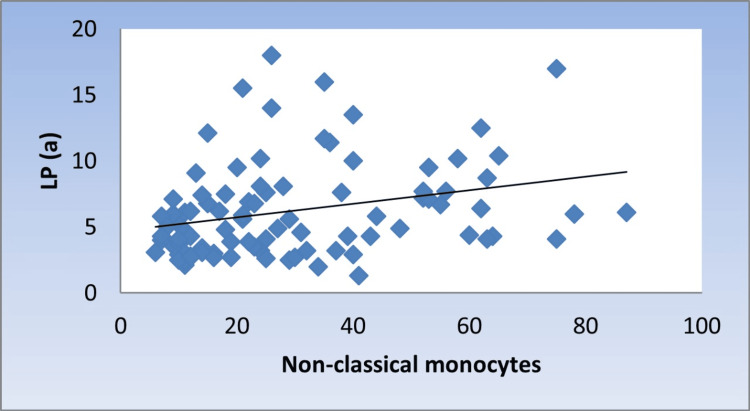
Scatter diagram representing positive correlation between non-classical monocytes and Lp(a) Lp(a): lipoprotein(a)

There was a significant moderate positive correlation between Lp(a) with monocyte counts, M/L index, and NCMs (r = 0.424, 0.376, 0.336, p < 0.001, <0.001, 0.001, respectively) (Figure 1, Figure 2, and Figure 3).

**Figure 4:**
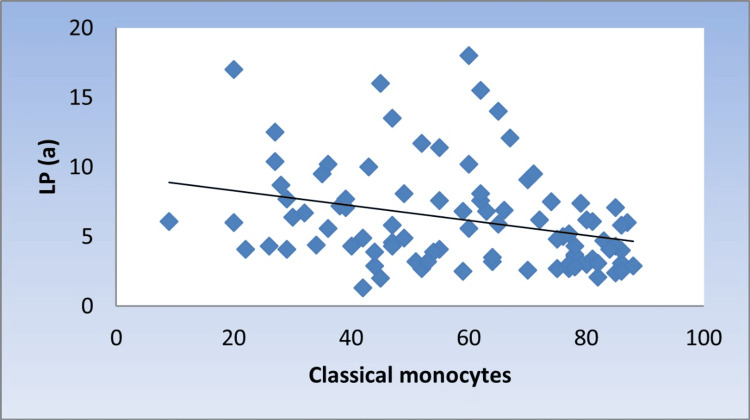
Scatter diagram representing negative correlation between classical monocytes and Lp(a) Lp(a): lipoprotein (a)

However, there was a significant moderate negative correlation with CMs (r =-0.346, p = 0.001) (Figure 4).

Meanwhile, there was no significant correlation with IMs (r = 0.145, p = 0.174).

Association between different variables and Lp(a) values less or more than 6.2 mg/dL

Patients were divided into two subgroups based on Lp(a) concentrations (greater than or less than 6.2 mg/dL). Patients with Lp(a) levels exceeding 6.2 mg/dL showed a significantly higher prevalence of positive CRP (p = 0.003), monocytes %, monocytes count, and M/L index (p = 0.001, 0.0003, 0.009, respectively), and a significantly higher median NCM (p = 0.005) compared to patients with Lp(a) levels below 6.2 mg/dL. No statistically significant differences were observed between the two groups for age, gender, IM subsets, and other blood picture parameters. In patients with Lp(a) values more than 6.2 mg/dL, triple-vessel lesions occurred in 60% of patients, which means that Lp(a) > 6.2 mg/dL was associated with the presence of triple-vessel coronary artery disease (CAD) (Table 2).

**Table 2:** Comparison between subjects with Lp(a) values less or more than 6.2 mg/dL HB: hemoglobin, PLT: platelets, WBCs: white blood cells, M/L index: monocyte-lymphocyte index, Lp(a): lipoprotein (a), CRP: C-reactive protein

	<6.2 mg/dL (n = 24)	≥6.2 mg/dL (n = 36)	p-value
Age (year)	47.5 ± 12	50.9 ± 11.5	0.186
Gender (male, female)	35(64.8), 19(35.2)	17 (47.2), 19 (52.8)	0.098
HB (g/dL)	13.45 ± 1.33	13.07 ± 2.02	0.285
WBCs (× 10^3^/µL)	6.25 (4-10.9)	7.15 (4-18)	0.104
PLT (× 10^3^/µL)	264 ± 79.8	263.8 ± 87.9	0.972
Monocytes (%)	7 (3-16)	9 (3-17)	0.001*
Lymphocytes (%)	33.48 ± 10.13	31.94 ± 7.46	0.438
Neutrophils (%)	54.43 ± 12.2	54.83 ± 8.93	0.864
M/L index	0.22 (0.08-0.41)	0.26 (0.08-0.77)	0.009
CRP positive (%)	20.4	50	0.003*
Classical (%)	67.5 (9-88)	55 (20-85)	0.010*
Intermediate (%)	10 (2-37)	11.5 (5-26)	0.157
Non-classical (%)	20 (6-87)	31.5 (9-75)	0.005*

Diagnostic accuracy of Lp(a)

**Figure 5:**
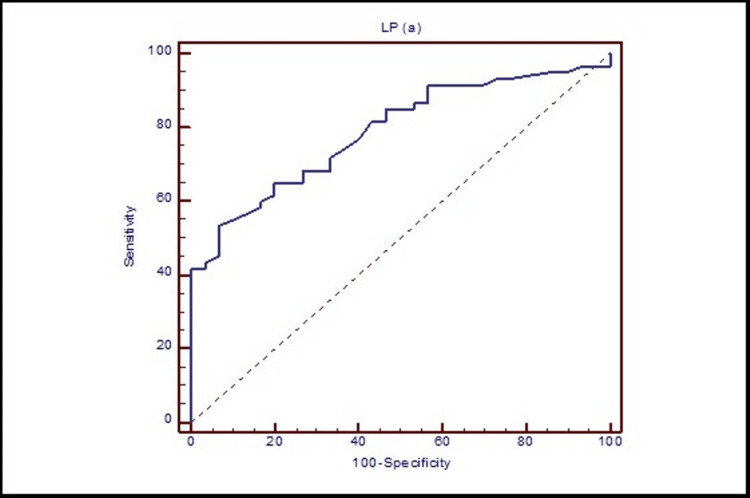
Receiver operating characteristic (ROC) curve of Lp(a) for diagnosis of atherosclerosis

ROC curve analysis of Lp(a) in the diagnosis of atherosclerosis is presented in Figure 5 and showed that the cutoff value of Lp(a) for differentiating between atherosclerosis and normal subjects was 6.2 mg/dL. At this cutoff value, the sensitivity, specificity, accuracy, PPV, and NPV were 53.3%, 93.3%, 66.6%, 94.1%, and 50% (area under the curve (AUC) 0.789, 95% CI 0.69, 0.868, respectively) (Figure 5).

Binary logistic regression analysis

Significant Predictors of Lp(a) Categories (<6.2 or ≥ 6.2 mg/dL)

A binary logistic regression model was constructed using Lp(a) categories (<6.2 or ≥ 6.2 mg/dL) as the dependent variable. The independent variables were gender, age, monocyte subsets, and the number of affected vessels. Model fitting was tested by several methods. First, the significant -2 log-likelihood test (-2 log-likelihood = 103.4, p = 0.013). Second, pseudo-R2 test results were as follows: Cox and Snell = 0.179, Nagelkerke = 0.242. The values of these tests indicate a good model fit. Results of the regression model showed that the presence of multiple affected vessels was the only significant predictor of Lp(a) categories. Subjects with multiple affected vessels are 0.159 folds less prone to have Lp(a) ≥ 6.2 mg/dL. In other words, subjects with multiple affected vessels are 6.3 folds more prone to have Lp(a) <6.2 mg/dL. (Please note that 6.3 is calculated as 1/0.159 = 6.3) (Table 3).

**Table 3:** Results of binary logistic regression analysis model for predictors of Lp(a) categories Lp(a): lipoprotein (a)

Variable	Regression coefficient (β)	Standard error (SE)	p-value	Odds ratio (OR)	95% CI
Gender (reference category: female)	0.836	0.499	0.094	2.306	0.876-6.132
Age	-0.001	0.024	0.951	0.999	0.953-1.046
Classical monocytes	0.093	0.458	0.840	1.097	0.447-2.694
Intermediate monocytes	0.079	0.457	0.863	1.082	0.442-2.648
Non-classical monocytes	0.124	0.457	0.786	1.132	0.462-2.772
Affected vessels (reference category: single)	-	-	-	-	-
Double	-0.598	0.670	0.372	0.550	0.148-2.042
Multiple	-1.840	0.673	0.006*	0.159	0.042-0.594

## Discussion

Lp(a)’s physiological role and its high atherogenicity mechanisms are not clear. It is stated that Lp(a) containing immune complexes interact with macrophages, leading to vessel wall inflammation [11]. Atherosclerosis is a complex disease characterized by lipid accumulation within the arterial wall and inflammation [12]. Innate immune cells, especially monocytes and macrophages, play a vital role in their initiation and progression through their influx into the vessel wall. A high circulating monocyte number is a high-risk factor for cardiovascular events [13,14]. Furthermore, increased NCMs in patients with high Lp(a) levels have been associated with an elevated risk of multi-vessel disease [12,15]. This study, for the first time, demonstrates the association between Lp(a) levels greater than or equal to 6.2 mg/dL and monocyte subsets.

In this study, similar to Xiang et al. and in contrast to Matei et al., gender and age were not significantly different from control [13,16]. Also, another study noted that males develop atherosclerotic plaques earlier, and plaques have a greater inflamed area [17].

Platelets have an important role in coronary thrombosis pathogenesis and atherogenesis. Similar to Matei et al., platelets had a significant difference between mild and severe atherosclerosis (p = 0.033) [16]. Studies have shown that platelet activity is different among populations, which can explain CVD by several mechanisms [18].

CRP is an early indicator of inflammatory conditions; it mediates tissue fibrosis in several CVDs [16]. In this study, a higher prevalence of positive CRP in the patient group than in the control group was found (p = 0.026). Also, there were significant differences among mild, moderate, and severe groups (p < 0.0001) [16]. This is similar to another study that found increased levels of CRP strongly predict thrombotic complications of atherosclerosis and are associated with CVD risk [19,20].

Lp(a) showed significantly higher median levels than the control group (p < 0.001). However, there was no significant difference in Lp(a) levels regarding the severity of atherosclerosis. This may explain the association between Lp(a) and coronary artery atherosclerosis, the high residual risk of myocardial infarction [21,22]. This supports the role of Lp(a) in improving risk assessment, which may be useful for primary prevention therapy decisions [23,24]. On the contrary, another study found that coronary atherosclerosis severity was correlated with the Lp(a) level [25]. 

In studies by Patel et al. and Idzkowska et al. [26,27], the median monocytic % in atherosclerosis patients was significantly higher than the control group (p = 0.045). Also, monocytic count is significantly higher in the severe atherosclerotic subgroup compared with mild and moderate subgroups (p = 0.008). This agrees with a study that observed that patients with low monocyte counts have lower survival rates [15], and a high monocyte count was independently associated with a better prognosis

In agreement with Xiang et al., who found a significant increase in intermediate and NCM subsets [13] in the patient group compared to the control group, a recent study showed that individuals with unstable angina had a higher number of circulating IMs. However, asymptomatic subjects and patients with smaller plaques had higher quantities of CMs [28], supporting the association between IMs and the occurrence of major cardiovascular events and increased mortality [26]. On the other hand, Xiang et al. [13] found no significant correlations between monocytes subsets and CA severity.

The M/L index (r = 0.376, p < 0.001), a new marker of systemic inflammation, is associated with CVD prognosis and independently associated with increased CVD mortality in atherosclerotic cardiovascular patients [29]. Additionally, another study suggested that monocyte count and MLR may be potential, cost-effective, and clinically available indicators of CVD risk [30].

Here, Lp(a) had a significant moderate positive correlation with monocyte count (r = 0.424, p < 0.001). Similarly, the increased monocyte count in CVD is related to lipid levels, primarily to low HDL-C, which promotes monocytosis [26]. A significant moderate positive correlation between Lp(a) and NCMs (r = 0.336, p = 0.001) was found, which is similar to a study that found that relative NCMs are associated with Lp(a) concentration (r = 0.18, p = 0.03). This supported the relationship between an increased Lp(a) level and multiple vessel atherosclerosis as Lp(a) causes endothelial dysfunction and damage through different mechanisms, one of them being increased non-classical CD14+CD16++ monocytes in patients with hyper Lp(a). This is due to their “innate” control of tissues to detect Lp(a)-damaged endothelium [31,32].

NCMs have pro-inflammatory properties through high cytokine-level production when stimulated [33]. This high level is involved in T-cell stimulation and proliferation [34,35]. The established association we found between NCM increase and triple-vessel disease in Lp(a) high cases indicated that the redistribution of monocytes from classical to “pro-inflammatory” subsets may be mediated by this atherogenic lipoprotein. Understanding monocytes’ regulation, differentiation, and functioning in the presence of Lp(a) disorders may define future therapeutic interventions aimed at activating or blocking immune system components.

Limitations of the study

Patients included in the study were admitted to a single center. Other patients from different centers should be included to confirm the results. The relation between the extent of peripheral atherosclerosis and the studied parameters was not assessed. Another limitation is that other monocytic markers, such as CD64, and markers of IMs like CCR2, CXCR1, and CCR5 should be assayed. These limitations should be considered while interpreting the results.

## Conclusions

The findings of this study revealed an association between Lp(a) concentration and the blood content of intermediate and NCMs, regardless of gender and age. The increased level of Lp(a) and the decreased quantity of CMs were associated with the severity of coronary atherosclerosis. The expansion of CD16+ monocytes (intermediate and non-classical) in the presence of hyper Lp(a) signiﬁcantly increased the risk of triple-vessel coronary disease.

Lp(a) was found to have no significant difference regarding the disease severity, while monocytic count and differentiated subsets were significantly affected with the disease progression. Thus, an increase in the percentage of circulating intermediate and NCM subsets can be regarded as an appropriate prognostic marker in patients with coronary artery atherosclerosis, while Lp(a) may have no prognostic value.
